# Biosorption of Pb (II) and Zn (II) from aqueous solution by *Oceanobacillus profundus* isolated from an abandoned mine

**DOI:** 10.1038/s41598-020-78187-4

**Published:** 2020-12-03

**Authors:** Wilson Mwandira, Kazunori Nakashima, Satoru Kawasaki, Allison Arabelo, Kawawa Banda, Imasiku Nyambe, Meki Chirwa, Mayumi Ito, Tsutomu Sato, Toshifumi Igarashi, Hokuto Nakata, Shouta Nakayama, Mayumi Ishizuka

**Affiliations:** 1 Faculty of Engineering, Hokkaido University, Kita 13, Nishi 8, Kita-Ku, Sapporo, 060-8628 Japan; 2 IWRM Centre/Geology Department, School of Mines, University of Zambia, P.O. Box 32379, Lusaka, Zambia; 3 Department of Mining, Metallurgical, and Materials Engineering, University of the Philippines Diliman, Quezon City, Philippines; 4 Graduate School of Veterinary Medicine, Hokkaido University, Kita 18, Nishi 9, Kita-Ku, Sapporo, 060-0818 Japan

**Keywords:** Biotechnology, Environmental sciences

## Abstract

The present study investigated biosorption of Pb (II) and Zn (II) using a heavy metal tolerant bacterium *Oceanobacillus profundus* KBZ 3-2 isolated from a contaminated site. The effects of process parameters such as effect on bacterial growth, pH and initial lead ion concentration were studied. The results showed that the maximum removal percentage for Pb (II) was 97% at an initial concentration of 50 mg/L whereas maximum removal percentage for Zn (II) was at 54% at an initial concentration of 2 mg/L obtained at pH 6 and 30 °C. The isolated bacteria were found to sequester both Pb (II) and Zn (II) in the extracellular polymeric substance (EPS). The EPS facilitates ion exchange and metal chelation-complexation by virtue of the existence of ionizable functional groups such as carboxyl, sulfate, and phosphate present in the protein and polysaccharides. Therefore, the use of indigenous bacteria in the remediation of contaminated water is an eco-friendly way of solving anthropogenic contamination.

## Introduction

The contamination of water bodies by heavy metals has resulted in increased research on how to remove such toxic pollutants from the environment. The most investigated heavy metals include chromium (Cr), cadmium (Cd), zinc (Zn), mercury (Hg), lead (Pb), nickel (Ni), and arsenic (As) because of the significant public health and environmental risks that they pose^1^. These heavy metals are introduced into the environment by human activities such as mining and agriculture^2^. Reverse osmosis, ion exchange, precipitation, and solvent extraction have been used to decontaminate wastewater before release into the natural environment; however, these technologies are either extremely expensive or not sufficiently effective^3^. The removal of heavy metals using biological materials has emerged as one of the most promising alternatives, as it does not induce secondary pollution and is cost-effective by avoiding the need for sludge disposal systems^4^. Biological materials employed for the removal of heavy metals from aqueous solutions include bacteria^5^, yeast^6^, algae^7^, fungi^8^, agricultural waste^4^ or wood waste^9^. The isolation and identification of many indigenously available biological materials are indispensable in sites heavily contaminated with heavy metals.

Heavy metal bioremediation can be classified as either bioaccumulation, a metabolically controlled process that requires the active uptake of heavy metal ions by living biomass, or biosorption, a non-metabolic process that passively binds metal cations onto non-living biomass^4^. Bioaccumulation requires energy for the uptake of metal ions and typically occurs through the interaction of metal ions and the cell wall. The second step is intracellular uptake, wherein metal ions penetrate the cell membrane and enter the cell to bind on the active sites provided by polysaccharides and proteins. Biosorption relies on the presence of functional groups in the cell wall and/or metabolites exported to the external area. Mechanisms of biosorption include ion exchange, complexation, precipitation, reduction, and chelation^10^.

Under harsh conditions in the presence of toxic heavy metals and antibiotics, bacteria commonly produce extracellular polymeric substances (EPS) as a protective response. Bacterial EPS are natural polymers of high molecular weight secreted by microorganisms into their environment^11^. Bacterial EPS typically contains polysaccharides with ionizable functional groups such as carboxyl, sulfate, and phosphate. These functional groups can be deprotonated to anionic species, which interact with cationic metal ions through electrostatic interactions, resulting in the immobilization of heavy metals within the EPS^12^. Biosorption through EPS has been extensively studied because of its efficient sequestration of toxic metals^10,13,14^. The metabolism-independent process of biosorption within the EPS is typically more favorable than bioaccumulation within the cell, which is metabolism-dependent^12^. Biosorption has thus received significant research attention. Despite the isolation of various types of bacteria, the isolation and characterization of indigenous bacteria are indispensable since they are environmentally acceptable and preserve the ecosystem in their locality as evidenced by studies that have used metal tolerant bacteria from industrial effluent^15^. The local population, soil, water, and food are contaminated with Pb (II) and Zn (II) in the study area at Kabwe Mine, Zambia, Africa, leading Kabwe to be labeled as one of the 10 most polluted places on Earth in 2013^16^.

Therefore, this study aims to assess the heavy metal removal by *Oceanobacillus profundus* KBZ 3-2, a strain isolated by our research group at the Kabwe Mine site, Zambia, Africa^17^. This bacterium was isolated when we investigated the biocementation of mine waste by immobilization using ureolytic bacteria by microbially induced calcium carbonate precipitation. The results from the investigation showed that mine waste was stabilized by indigenous bacteria. Additionally, during the characterizing of the bacteria isolated from the site, we found out that *O. profundus* KBZ 3-2 had a higher biosorption capacity and was worth investigation for heavy metal removal. This study investigated the removal of heavy metals by isolated bacteria in single metal ion batch experiments in a solution prepared to imitate the environment at the abandoned lead–zinc mine at Kabwe, Zambia. The Pb (II) and Zn (II) metal ions were selected for investigation because both pollutants have caused significant health problems to communities near the site^18^. Moreover, the mechanism of biosorption was discussed and especially focused on the dominant factor affecting biosorption.

## Materials and methods

### Bacterial strain and chemical reagents

The mine waste was exported from the abandoned Kabwe Mine, Zambia under approval No. RCT 7686229, and the import was permitted by Plant Protection Station, Ministry of Agriculture, Forestry and Fisheries, Japan under the approval No. 29-836. The bacterial strain *O. profundus* KBZ 3-2 used in this study was isolated from the mine waste when screening for ureolytic bacteria for the solidification of sand^17^. The same methodology described in our previous study was used to screen the bacterium^17^. Chemical reagents used in this study were obtained from Wako Pure Chemical Industries Ltd., Tokyo, Japan, otherwise mentioned.

### Growth of bacteria in the presence of Pb (II)/Zn (II)

After preculturing *O. profundus* KBZ 3-2, the bacteria were cultured in 100 mL of LB medium containing PbCl_2_ or ZnCl_2_ with different concentrations ranging from 0–50 mg/L for 24 h. Cell growth was monitored by checking the turbidity of the culture with a UV–vis spectrophotometer at 600 nm (OD_600_).

### Effect of pH on biosorption of Pb (II)/Zn (II)

The bacteria were inoculated in a 100 mL LB medium containing 20 mg/L of PbCl_2_ or 2 mg/L of ZnCl_2_ at different pH ranging from 2 to 9 and adjusted by HNO_3_ or NaOH. After cultivation at 30 °C at 160 rpm for 24 h, the cell culture was centrifuged at 8000 × *g* at 4 °C for 10 min to separate the supernatant and precipitate. The heavy metal concentrations of Pb or Zn in the supernatant were measured by inductively coupled plasma atomic emission spectroscopy (ICP-AES) (ICPE-9820, Shimadzu Corporation, Kyoto, Japan). The biosorption efficiency of heavy metals by the bacteria was calculated as (*C*_i_ – *C*_f_)/*C*_i_ × 100 (%), where *C*_i_ is the initial concentration, and *C*_f_ is the final concentration of metal ions.

### Localization of Pb (II) and Zn (II) in bacterial cells

**Scheme 1:**
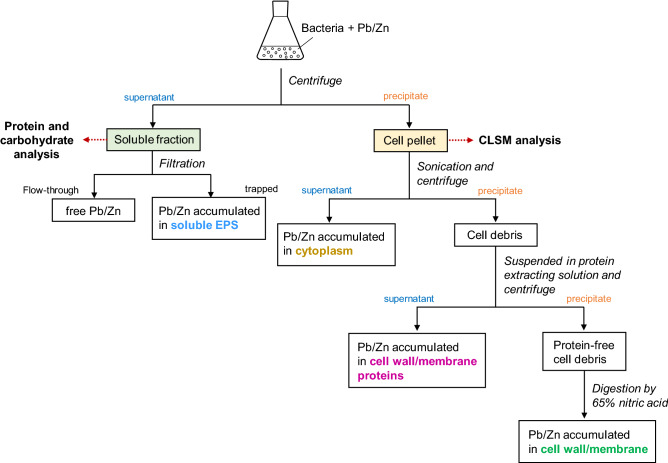
Flowchart for the analytical procedure for the determination of the distribution of heavy metal in different cellar parts.

The localization of Pb (II) and Zn (II) in cellular parts was determined according to the methodology reported by Sheng, et al.^19^ with some modifications and has been illustrated in Scheme 1. Briefly, cells were cultured in 100 mL of LB medium containing 20 mg/L of Pb or 2 mg/L of Zn at 30 °C at 160 rpm for 24 h and were harvested by centrifuge at 8000 × *g* for 10 min. The supernatant was filtered by a 0.45 μm membrane filter, where EPS and EPS-bound metal ions were trapped and free metal ions passed through. The concentration of Pb (II) or Zn (II) in the filtrate was measured by ICP-AES. The cell pellets were ultrasonicated at 30 kHz for 5 min (Vibra-Cell VCX 130, Sonics & Materials, Inc., Newtown, USA) in 5 mL Tris–HCl (10 mM, pH 8.0), followed by centrifugation at 8000 × *g* for 10 min to separate the supernatant (cell-free extract: cytoplasm) and precipitate (cell debris: cell wall/membrane). The supernatant was thereafter collected for analysis to determine the cytoplasmic water-soluble Pb (II) or Zn (II). The precipitate, cell debris, was then suspended in protein extracting solution (5% Sodium Dodecyl Sulfate: SDS, 10 mM Tris–HCl, pH 8), followed by centrifugation at 8000 × *g* for 10 min. The resulting supernatant was used for the determination of metal ions accumulated in cell wall/membrane proteins, and the obtained precipitate was digested by 65% nitric acid to quantify the Pb (II) or Zn (II) accumulated in cell wall/membrane components. The concentration of Pb (II) or Zn (II) accumulated in EPS was calculated by subtracting the metal concentration in the other factions from the initial concentration. Meanwhile, protein and carbohydrate contents in a supernatant (soluble fraction) after 24 h cell cultivation were measured. The protein content in the supernatant was determined by the Bradford protein assay with bovine serum albumin as the standard, and the total carbohydrate content was determined by the phenol sulfuric acid method as previously described^20^.

### Confocal laser scanning microscopy (CLSM) analysis

The bacteria cells were cultured in 100 mL LB medium for 24 h in the presence of 20 mg/L of Pb (II) and collected by centrifuge at 8000 × *g* for 10 min, followed by suspension in saline buffer. Three different staining dyes were sequentially added to the cell suspension to stain the DNA (DAPI Nucleic Acid Stain, Molecular probes, Invitrogen), EPS (Wheat Germ Agglutinin, Alexa Fluor 633 Conjugate, Molecular Probes, Invitrogen), and Pb (II) (Leadmium Green AM Dye, Molecular Probes, Invitrogen). The samples were analyzed with a CLSM (Nikon A1 and Ti-E) equipped with a Plan Apo VC × 60 objective lens (NA 1.40, Nikon).

## Results and discussion

### Effect of Pb (II) and Zn (II) on bacterial growth

**Figure 1:**
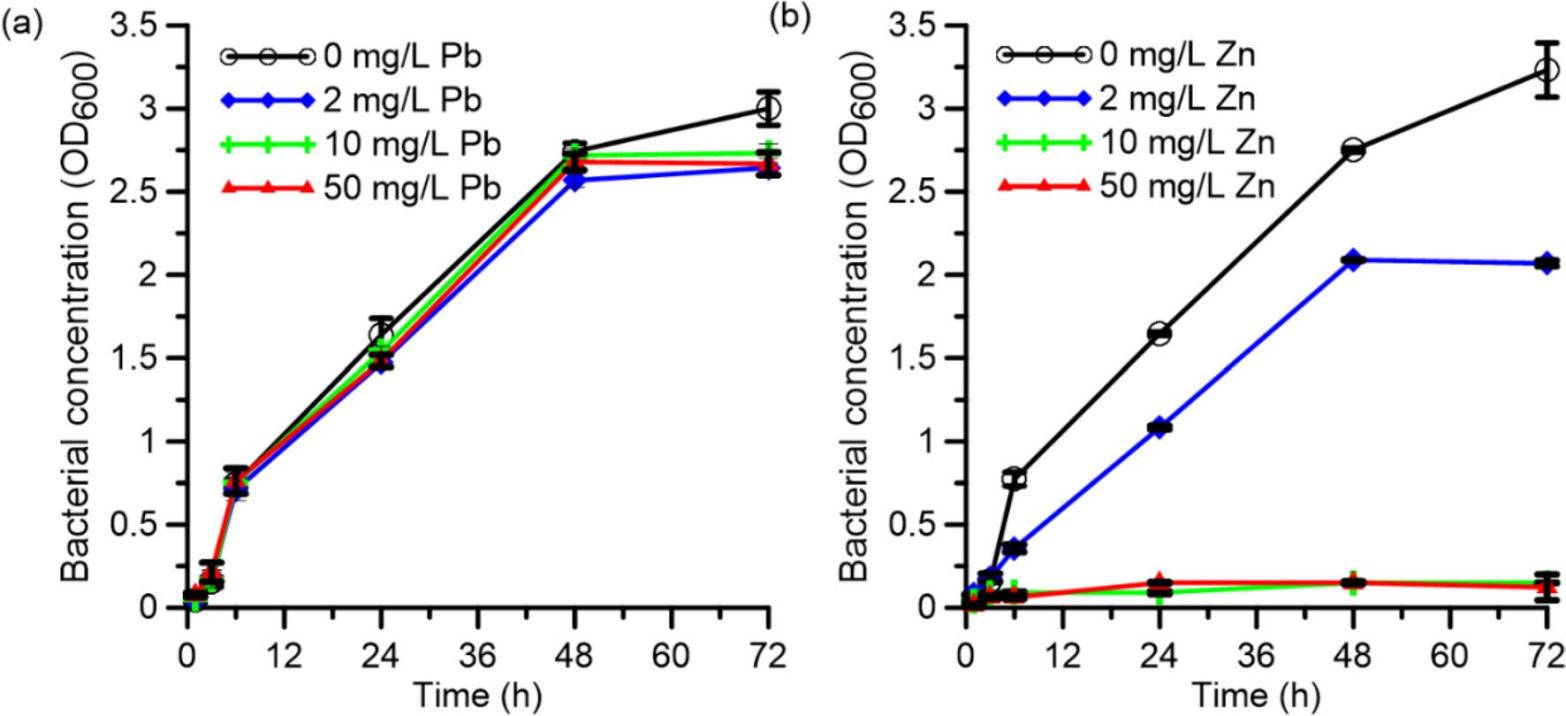
Microbial growth of *O. profundus* KBZ 3-2 in the presence of **(a)** Pb (II) and **(b)** Zn (II) with different concentrations.

Bacterial growth under exposure to the heavy metal ions of interest was assessed to gauge the applicability of the strain for bioremediation. Figure 1 shows the effect of Pb (II) and Zn (II) on the growth of *O. profundus* KBZ 3-2 in different concentrations. The bacterial cells grew well in all the tested concentrations of Pb (II), demonstrating resistance to Pb (II) contamination up to 50 mg/L. However, the bacteria were less tolerant of Zn (II), displaying growth only in 2 mg/L Zn (II). This result indicates that the bacteria can be used for bioremediation of Pb (II) and Zn (II), albeit at a lower concentration for the latter. Inefficient biosorption of Zn (II) at higher Zn(II) concentrations occurs because Zn (II) is a trace element required for the growth of heterotrophic bacteria^21^, such as *O. profundus.* However, at higher concentrations, Zn (II) hinders the survival of the bacteria; this is consistent with previous results that Zn (II) is a highly reactive divalent metal ion and that it would readily displace metal ions required for bacterial growth^22^. Moreover, Zn (II) is a known antibacterial agent because it is a strong oxidative agent that causes cell membrane disruption, leading to cell death^22^.

### Effect of pH on adsorption

**Figure 2:**
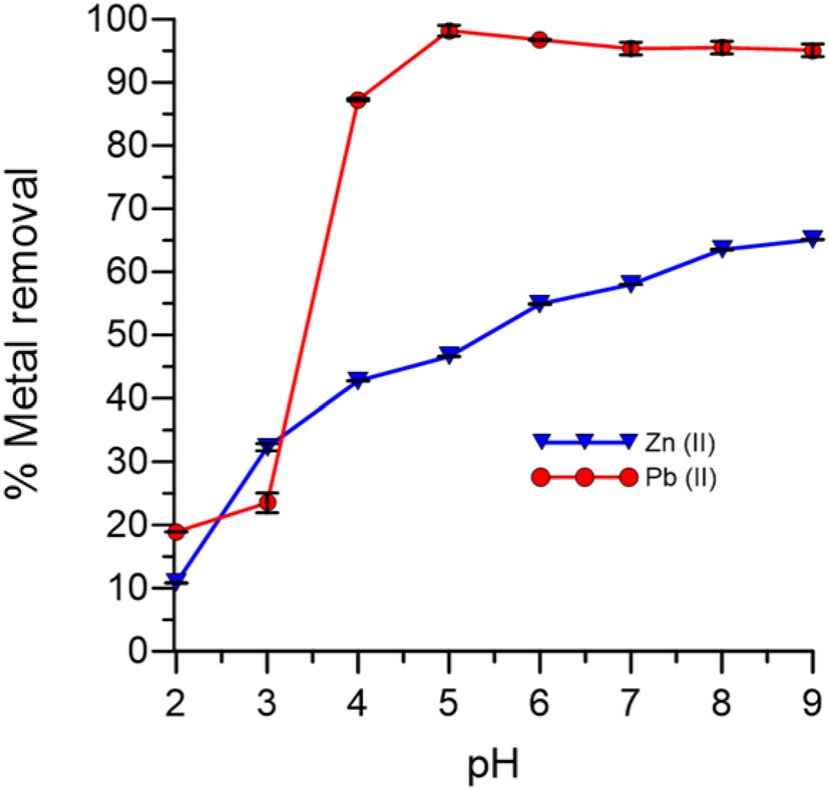
Effect of pH on biosorption by *O. profundus* KBZ 3-2.

The pH of the solution is among the most important parameters in biosorption because the initial pH of the solution is a key factor that affects the metal speciation, metal solubility, and the dissociation of the functional groups^23,24^. Figure 2 shows the effect of pH on heavy metal removal efficiency. The removal percentage of metal ions was as low as 18% for Pb (II) and 12% for Zn (II) at pH 2. The maximum removal percentage for Pb (II) was 97% at an initial concentration of 50 mg/L whereas maximum removal percentage for Zn (II) was 54% at an initial concentration of 2 mg/L obtained at pH 6. This low removal percentage at low pH was attributed to the protonation of functional groups, which were responsible for metal ion adsorption^22^. Similar findings by earlier investigators have also attributed lower biosorption efficiency to protonation or poor ionization of functional groups at low pH, resulting in a weak complex affinity of the metal ions^25,26^. On the other hand, at higher pH values, the binding efficiency increased because the functional groups were negatively charged, thereby facilitating biosorption of the metal cations through ion exchange and metal chelation complexation. Additionally, the resulting effective pH for biosorption is consistent with the data obtained from bacteria in the Bacillaceae family, which showed an effective pH range of 5–9^22^. A sharp increase in biosorption was observed in Pb and a gradual increase for Zn was probably caused by the gradual exhaustion of active sites for the biosorption of zinc ions. This trend has been observed in previous investigation^5^. Table 1 represents a comparison between *O. profundus* KBZ 3-2 with other biosorbent materials. The results show the *O. profundus* KBZ 3-2 can be used as an effective biosorbent.

**Table 1:** Comparison between *O. profundus* KBZ 3-2 with other biosorbents.

Metal ion	Biosorbent	Metal ion conc	Adsorption efficiency (%)	Ref
Pb	*Pseudomonas* sp.	1 mg/L	87.9	^27^
	*S. maltophilia*	39.6 mg/L	96	^28^
	*B. iodium*	100 mg/L	87	^29^
	*O. profundus* KBZ 3–2	50 mg/L	97	This study
Zn	*S. maltophilia*	20.6 mg/L	96	^28^
	*Pseudomona*s sp.	1 mg/L	49.8	^27^
	Zinc sequestering bacterium VMSDCM	0.21 mol/g of biomass		^5^
	*O. profundus* KBZ 3–2	2 mg/L	54	This study

### Localization of Pb (II) and Zn (II) in a cell

**Figure 3:**
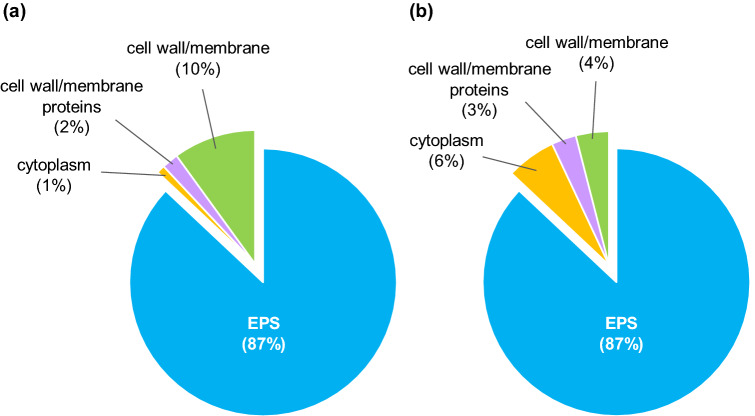
Distribution of **(a)** Pb(II) and **(b)** Zn(II) in different cellular parts of *O. profundus* KBZ 3-2.

We found there was no Pb/Zn left in the filtrate after filtration of the supernatant of cell culture added with Pb/Zn, which indicates heavy metal ions were totally trapped by the bacteria. The localization of metal ions in the different cellular parts of the bacteria was investigated to understand the possible mechanism of Pb (II) and Zn (II) accumulation in the bacteria cells. The percentages of metal ions in (1) soluble EPS, (2) the cytoplasm, (3) cell wall/membrane proteins, and (4) the cell wall/membrane of *O. profundus* KBZ 3-2 are shown in Fig. 3. The highest concentration for both elements was in soluble EPS (87%), which is produced in the soluble phase by bacteria as a defense mechanism against heavy metal contamination^22^. Pb (II) accumulation in other parts was determined in the cytoplasm (1%), cell wall/membrane proteins (2%), and the cell wall/membrane (10%). Zn (II) was found in the cytoplasm (6%), cell membrane/wall proteins (3%), and the cell wall/membrane (4%). For both heavy metals, biosorption would predominantly be metabolism-independent since it primarily occurred on the exterior of the cell, EPS. We can conclude that metabolism-dependent biosorption has a minor role^30^.

The amount of cytoplasmic Zn (II) in Fig. 3b was higher than that of Pb (II) in Fig. 3a, probably because Zn (II) is used for microbial metabolic processes in trace amounts. The formation of EPS-Pb could have prevented the entry of Pb into the cell; hence a small amount of Pb (II) was detected inside the cell. EPS contains different carbohydrates and their derivatives^31^, which are usually polyanionic because of the functional groups. This allows biosorption through mechanisms such as ion exchange and metal complexation^32^. Thus, we propose that the dominant mechanism of Pb (II) and Zn (II) biosorption by *O. profundus* KBZ 3-2 occurs by soluble EPS being actively secreted by a bacterium to defend itself from toxic ions, thereby binding Pb (II) and Zn (II) ions from solutions through metal chelation-complexation and consequently removing Pb (II) and Zn (II) through metabolism-independent process biosorption. Bioaccumulation also occurred, albeit at a significantly lesser degree, as evidenced by the presence of heavy metals within the cell.

### Composition of EPS released by the bacteria

**Figure 4:**
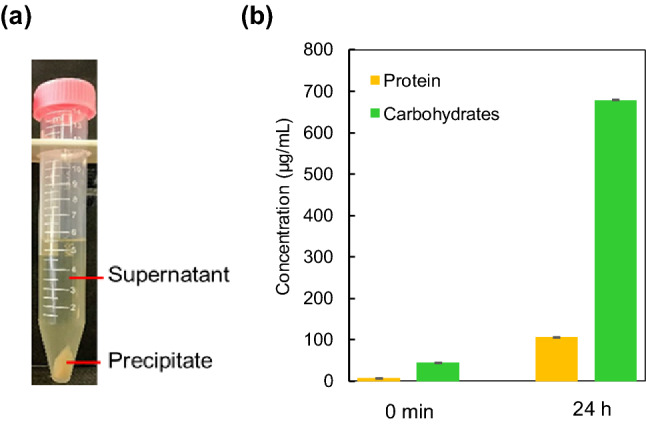
Analysis of *O. profundus* KBZ 3-2 cultured in 20 mg/L Pb for 24 h; **(a)** image of centrifuged culture, **(b)** quantitative determination of proteins and carbohydrates in soluble EPS.

**Figure 5:**
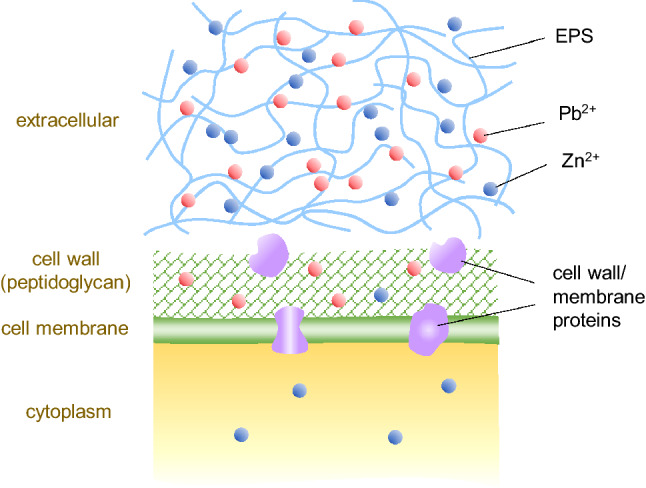
Representative diagram for biosorption mechanism for Pb(II) and Zn(II).

Figure 4a shows the photo of a centrifuged bacterial suspension-cultured for 24 h in the presence of 20 mg/L Pb. We observed the supernatant containing soluble EPS and the precipitates containing bacterial cells. The composition of soluble EPS was analyzed over time in terms of proteins and carbohydrates (Fig. 4b), because it is the main component responsible for metal sequestration. Typically, EPS consists of water, protein, polysaccharides, nucleic acid, uronic acid, and humic acid^33–35^. The EPS had 105 µg/L of protein and 679 µg/L of carbohydrates; these components are primarily responsible for the biosorption of Pb (II) and Zn (II) through metal chelation-complexation because they contribute to the negatively charged carboxyl group (–COO^–^), sulfate group (–SO_3_^–^), and phosphate group (–PO_3_^–^). This result is consistent with the characteristic of a bacterium having defensive mechanisms for Pb (II). Previous studies have also shown that bacterium can survive in the conditions with high heavy metal exposure by developing resistance and could be isolated and used for the bioremediation of Zn (II), Cd (II), Cu (II), and Pb (II)^27,31,32^. A representative diagram for the bacterial biosorption mechanism is depicted in Fig. 5.

### CLSM analysis

**Figure 6:**
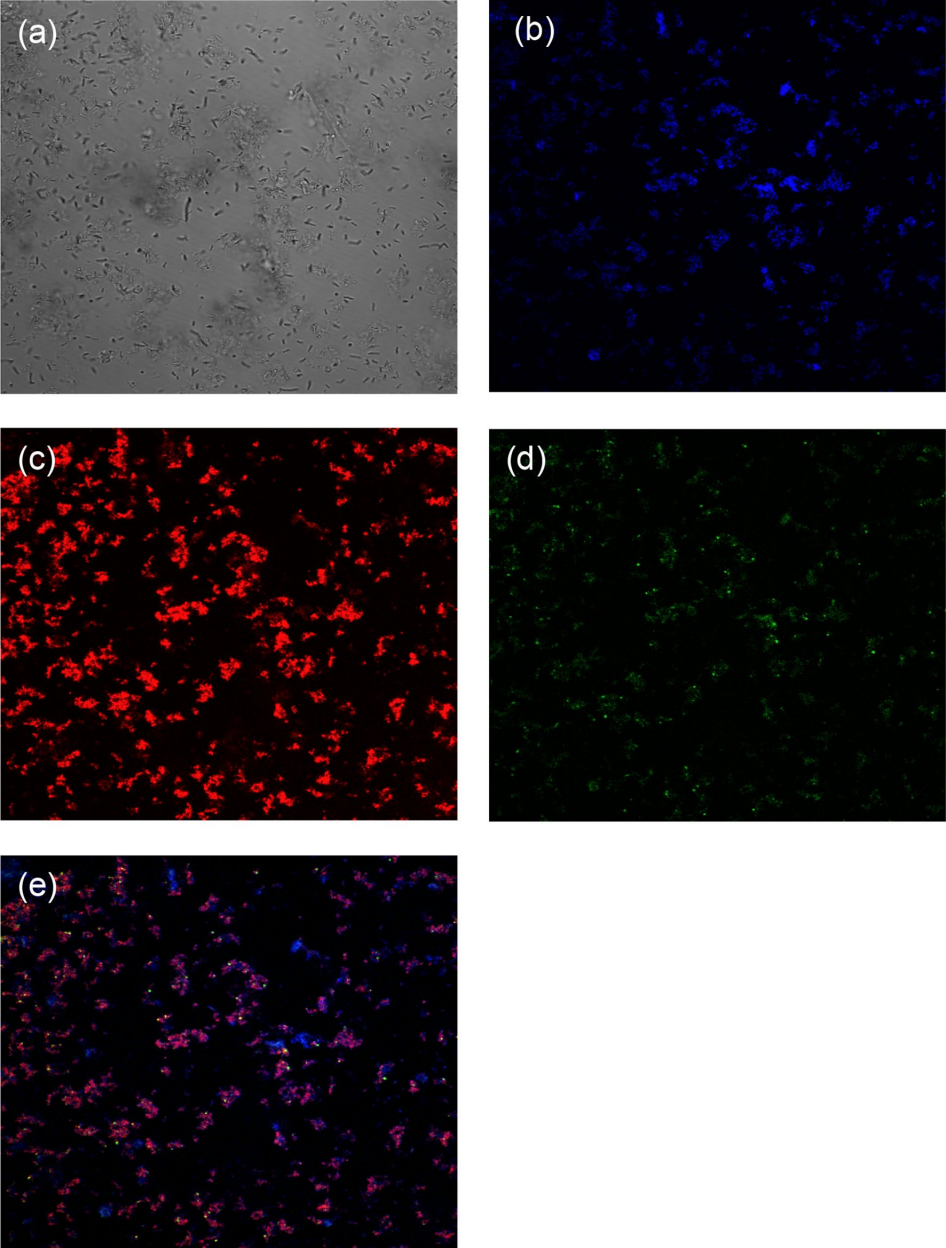
Confocal images of *O. profundus* KBZ 3-2 cultured in 20 mg/L Pb for 24 h; **(a)** bright-field image, **(b)** bacterial cells stained by DAPI (blue), **(c)** EPS stained by Alexa Fluor 633-conjugated agglutinin (red), **(d)** Pb (II) stained by Leadmium Green AM Dye (green), **(e)** overlay image of **(b–d**).

As indicated in the study methods, we conducted a CLSM analysis of grown cells in the presence of 20 mg/L Pb (II) to further confirm the native associations of cell-EPS-metal ion aggregates. Most of the EPS was found in the supernatant after centrifugation, some EPS would be staying around the cells. Figure 6 shows the CSLM images of the bacterial suspension, showing the bright-field image (a), the bacterial cells (b, in blue), EPS (c, in red), and Pb (II) (d, in green). The correlation and visualization of each component (bacterial cells, EPS, and Pb (II)) confirm the adsorption of Pb ions onto the cells and their surroundings. Since the cells were surrounded by the remaining EPS, which was secreted by cells, the protein and carbohydrates in EPS would be responsible for the heavy metal detoxification via complexation with metal ions. Consequently, EPS has an important role in the adsorption of different toxic heavy metals, which is consistent with the previous report^13^.

## Conclusion

The study demonstrated that *O. profundus* KBZ 3-2 isolated from Pb–Zn contaminated soil at the Kabwe Mine site in Zambia was capable of removing Pb (II) and Zn (II) from water. The proposed mechanism of biosorption was through chelation-complexation on the present functional groups of EPS excreted by the bacteria. *O. profundus* KBZ 3-2 was a highly efficient biosorbent and had high potential in upscaling its biosorption purpose for bioremediation at Kabwe Mine site as an eco-friendly solution to contaminated site restoration. This is recommended for the cleaning of contaminated water from the mine site.
